# Effect of collagen crosslinkers on sodium hypochlorite treated dentin bond strength: a systematic review and meta-analysis

**DOI:** 10.3389/fbioe.2025.1547158

**Published:** 2025-04-09

**Authors:** Weiqing Zhou, Shuting Feng, Xiaojun Chu, Shuaimei Xu, Xiongqun Zeng

**Affiliations:** ^1^ Department of Endodontics, Stomatological Hospital, School of Stomatology, Southern Medical University, Guangzhou, Guangdong, China; ^2^ Guangzhou Haizhu District Hospital of Stomatology, Guangzhou, Guangdong, China

**Keywords:** dental bonding, tensile strength, cross-linkers, sodium hypochlorite, meta-analysis, systematic review

## Abstract

**Introduction:**

The bond strength (BS) between composite resin and dentin is a crucial factor determining the long-term success of restorations. Sodium hypochlorite (NaOCl), a frequently used root canal irrigating agent, has been demonstrated to notably influence the properties of dentin, thereby affecting the BS. Moreover, the application of collagen crosslinkers has become a potential approach to improve the stability of the resin-dentin bond. Nevertheless, the effect of collagen crosslinkers on the bond strength (BS) between sodium hypochlorite (NaOCl) treated dentin and composite resin remains a topic of contention, and there is a lack of in-depth understanding in the existing literature. The purpose of this systematic review and meta-analysis was to analyze the current literature on the effect of collagen crosslinkers on the BS between sodium hypochlorite treated dentin and composite resin.

**Methods:**

Databases including PubMed, EMBASE, Cochrane library, Scopus, Web of Science and SinoMed were searched. *In vitro* studies reported the effect of crosslinking agents on NaOCl-treated dentin BS were included. The reference lists of studies included via databases were manually searched for more studies that fulfilled the inclusion criteria. The initial search yielded 1,538 studies, and subsequent screening resulted in the inclusion of 14 studies. Most of studies (78.6%, 11/14) were categorized as having a “low” risk of bias. The studies included in the meta-analysis employed a range of cross-linking agents, including ethylenediaminetetraacetic acid (EDTA), phytic acid (IP6), proanthocyanidin (PA), rosmarinic acid (RA) and sodium ascorbate (SA). Subgroup comparisons were performed according to NaOCl exposure duration. Studies treated with different concentration of NaOCl were analyzed separately.

**Results:**

For dentin exposed less than 1 min or NaOCl at lower concentration, significant positive effect cannot be observed when using collagen crosslinkers. For dentin exposed more than 1 min in NaOCl at concentrations greater than 2.5%, EDTA, PA and SA were observed to significantly improve the BS. RA is proved effective in improving the BS of dentin exposed to high concentrations NaOCl within a shorter duration. Current evidence is insufficient to conclude that IP6 has a positive effect in NaOCl-treated dentin bonding performance.

**Conclusion:**

The effect of collagen crosslinkers on the BS of NaOCl treated dentin was influenced by the concentration of NaOCl and the duration of exposure.

## 1 Introduction

In modern dentistry, the long-term success of dental restorations highly depends on the bond strength (BS) between composite resins and dentin. A strong and stable bond not only ensures restoration functionality but also prevents complications like secondary caries and debonding. However, achieving and maintaining optimal BS is challenging due to multiple influencing factors. One such factor is sodium hypochlorite (NaOCl), a commonly used root canal irrigant in clinical practice.

Sodium hypochlorite (NaOCl) is renowned for its potent bactericidal properties, which enable it to effectively eliminate microorganisms colonizing the root canal system and its walls. Additionally, it can neutralize bacterial toxins and dissolve both live and necrotic pulp tissue. Nevertheless, mounting evidence indicates that NaOCl may have adverse effects on dentin. Research has shown that its application can lead to the decomposition of collagen within dentin, thereby disrupting the formation of the mixed layer. Consequently, the adhesive is unable to effectively penetrate the collagen fiber network or dentin tubules (Carrilho et al., 2009; Cai et al., 2023). Moreover, NaOCl chlorination generates oxidation radicals that react with the monomer radicals generated during the polymerization of methacrylate monomers, as well as the initiator and radicals in the bonding system. the concentration of free radicals in the growth chain is diminished, resulting in premature chain termination and subsequently influencing the polymerization reaction during the bonding process. It has been demonstrated in numerous studies that NaOCl markedly diminishes the BS of dentin (Morris et al., 2001; Farina et al., 2011; Dikmen, 2015; Pimentel Correa et al., 2016; Haralur et al., 2017; Li et al., 2022; Xu et al., 2022).

Collagen fibers are pivotal in anchoring the composite resin to the dentin surface. The bio-stability of both the underlying demineralized collagen and infiltrated resin is considered one of the most important factors for success of dental restorations (Breschi et al., 2008; Breschi et al., 2018). The enzymatic degradation of bare collagen and the hydrolytic precipitation of resin within the mixed layer are the primary factors influencing the durability of resin dentin bonding (Ricucci and Siqueira, 2011; Prasansuttiporn et al., 2012). The BS is mainly provided by the penetration of the resin into the demineralized dentin, which forms a mechanical inlay force. Incomplete resin monomer penetration results in the exposure of collagen fibers and subsequent degradation of these fibers under the influence of water, enzymes, bacteria, stress, temperature and other factors (Betancourt et al., 2019). This eventually leads to the failure of adhesive bonded restorations (Carvalho et al., 2005). To address these challenges and enhance the stability of collagen fibers in the bonding interface, the use of cross-linking agents has been proposed, which can promote the inter or intramolecular cross- linking of collagen molecules (Shafiei et al., 2023), thereby maintaining the integrity of the bonding interface.

This systematic review and meta-analysis aim to explore the effect of crosslinkers on BS between NaOCl-treated dentin and composite resins. Three types of crosslinkers are investigated. The synthetic chelating agent-based crosslinker, ethylenediaminetetraacetic acid (EDTA), chelates metal ions to form bridging complexes (Lanigan and Yamarik, 2002). Natural polyphenolic crosslinkers such as proanthocyanidin (PA), phytic acid (IP6), and rosmarinic acid (RA) form coordination or covalent bonds with the polar groups of metal ions or biomacromolecules via their phenolic hydroxyl groups or phosphoric acid groups (Choi et al., 2016; Ge et al., 2018; Visan and Angelescu, 2023). The organic acid salt crosslinker, sodium ascorbate (SA), generates active intermediates through redox reactions to facilitate covalent crosslinking (Boyera et al., 1998).

Despite it is widely recognized that cross-linking agents contribute to the bonding of composite resins to dentin, (Anumula et al., 2022; Hardan et al., 2022; Chen et al., 2023), their the impact on NaOCl-treated dentin remains a topic of contention. Given the lack of previous relevant meta-analyses, this study intends to fill this knowledge gap. The null hypothesis tested was that collagen crosslinkers have no effect on the BS of NaOCl-treated dentin during bonding procedures. Through a comprehensive search of relevant databases, strict inclusion and exclusion criteria, and data extraction and analysis, this study anticipates providing valuable insights into the role of crosslinkers in improving the bond strength between NaOCl-treated dentin and composite resins, which may ultimately contribute to more successful dental restorations.

## 2 Materials and methods

This systematic review was conducted following the Preferred Reporting Items for Systematic Reviews and Meta-Analysis (PRISMA) statement (Page et al., 2021) and has been registered in PROSPERO, number CRD42023451577. The research question of this review was: “Does crosslinking agents improve the BS of composite resin to NaOCl-treated dentin?” It is established on the PICOS framework: population (dentin substrate); intervention (use of crosslinking agents); control (adhesive application of NaOCl-treated dentin without the use of a crosslinking agents); outcome (BS); study design (*in vitro* studies).

### 2.1 Search strategy

The literature search was performed by two independent reviewers (ZW and FS) and six electronic databases (PubMed, EMBASE, Cochrane library, Scopus, Web of Science and SinoMed) were screened to identify relevant manuscripts that could be included. Gray literature, encompassing theses, dissertations, and preprints, has been scrupulously searched as well. No publication year or language limit was used, and the database search was extended until 30th June 2024. Reference lists of the collected studies were also manually searched for additional relevant studies that met the inclusion criteria. Search strategy were detailed in Table 1.

**TABLE 1:** PubMed search strategy.

Number	Search strategy	Results
#1	“cross linking reagents” [MeSH Terms] OR “cross linking reagents” [Title/Abstract] OR “crosslinking reagent” [Title/Abstract] OR “cross linking reagent” [Title/Abstract] OR “crosslinking reagents” [Title/Abstract] OR “cross linking” [Title/Abstract] OR “Cross-linkers” [Title/Abstract] OR “Crosslinking” [Title/Abstract] OR “Cross-links” [Title/Abstract] OR “cross linking” [Title/Abstract] OR “cross linking agents” [Title/Abstract] OR “dentin collagen” [Title/Abstract] OR “cross linking agent” [Title/Abstract] OR “Crosslink” [Title/Abstract] OR “cross linker” [Title/Abstract]	99,607
#2	“edetic acid” [MeSH Terms] OR “edetic acid” [Title/Abstract] OR “EDTA” [Title/Abstract] OR “ethylenedinitrilotetraacetic acid” [Title/Abstract] OR “Glutaral” [MeSH Terms] OR “Glutaral” [Title/Abstract] OR “Glutardialdehyde” [Title/Abstract] OR “Glutaraldehyde” [Title/Abstract] OR “carbodiimides” [MeSH Terms] OR “carbodiimides” [Title/Abstract] OR “Riboflavin” [MeSH Terms] OR “Riboflavin” [Title/Abstract] OR “vitamin g” [Title/Abstract] OR “vitamin b2” [Title/Abstract] OR “Proanthocyanidins” [MeSH Terms] OR “Proanthocyanidins” [Title/Abstract] OR “epigallocatechin gallate” [Title/Abstract] OR “epigallocatechin-3-gallate” [Title/Abstract] OR “epigallocatechin-3-O-gallate” [Title/Abstract] OR “EGCG” [Title/Abstract] OR “genipin” [Title/Abstract] OR “Curcumin” [MeSH Terms] OR “Curcumin” [Title/Abstract] OR “Tannins” [MeSH Terms] OR “Tannins” [Title/Abstract] OR “tannic acid” [Title/Abstract] OR “Hesperidin” [MeSH Terms] OR “Hesperidin” [Title/Abstract]	153,610
#3	#1 OR #2	244,241
#4	“dentin” [MeSH Terms] OR “dentin” [Title/Abstract] OR “Dentins” [Title/Abstract] OR “Dentine” [Title/Abstract] OR “Dentines” [Title/Abstract]	38,386
#5	“sodium hypochlorite” [MeSH Terms] OR “sodium hypochlorite” [Title/Abstract] OR “hypochlorite sodium” [Title/Abstract] OR “Clorox” [Title/Abstract] OR “Antiformin” [Title/Abstract] OR “NaOCl” [Title/Abstract] OR “NaClO” [Title/Abstract] OR “hypochlorite sodium” [Title/Abstract]	11,941
#6	“tensile strength” [MeSH Terms] OR “tensile strength” [Title/Abstract] OR “shear strength” [MeSH Terms] OR “shear strength” [Title/Abstract] OR “microtensile strength” [Title/Abstract] OR “microshear strength” [Title/Abstract] OR “shear bond strength” [Title/Abstract] OR “bond strength” [Title/Abstract] OR (“Bongding” [All Fields] AND “performance” [Title/Abstract]) OR “bonding effectiveness” [Title/Abstract] OR “Bond” [Title/Abstract] OR “dental bonding” [MeSH Terms] OR “dental bonding” [Title/Abstract] OR “adhesives” [MeSH Terms] OR “adhesives” [Title/Abstract] OR “adhesion” [Title/Abstract] OR “dentin bonding agents” [MeSH Terms] OR “dentin bonding agents” [Title/Abstract] OR “dentin bonding agent” [Title/Abstract] OR “dentin bonding agent” [Title/Abstract]	486,554
#7	(#3) AND (#4) AND (#5) AND (#6)	338

### 2.2 Study selection

Studies were imported into NoteExpress (v4.0.0.9855). Two of the review authors (ZW and FS) independently assessed the titles and abstracts of all the studies. Full texts of potentially relevant studies were achieved after screening. A third reviewer (CX) was inquired for decision if an agreement could not be achieved. The inclusion criteria were as follows: (1) Evaluated the BS to NaOCl-treated dentin and composite resin; (2) Reported the effect on BS of the use of a crosslinking agent prior to the application of adhesive system (*in vitro*, *in vivo*, or both); (3) Included a control group where a crosslinking agent was not used; (4) Reported the micro tensile BS or shear bong strength in MPa.

**FIGURE 1:**
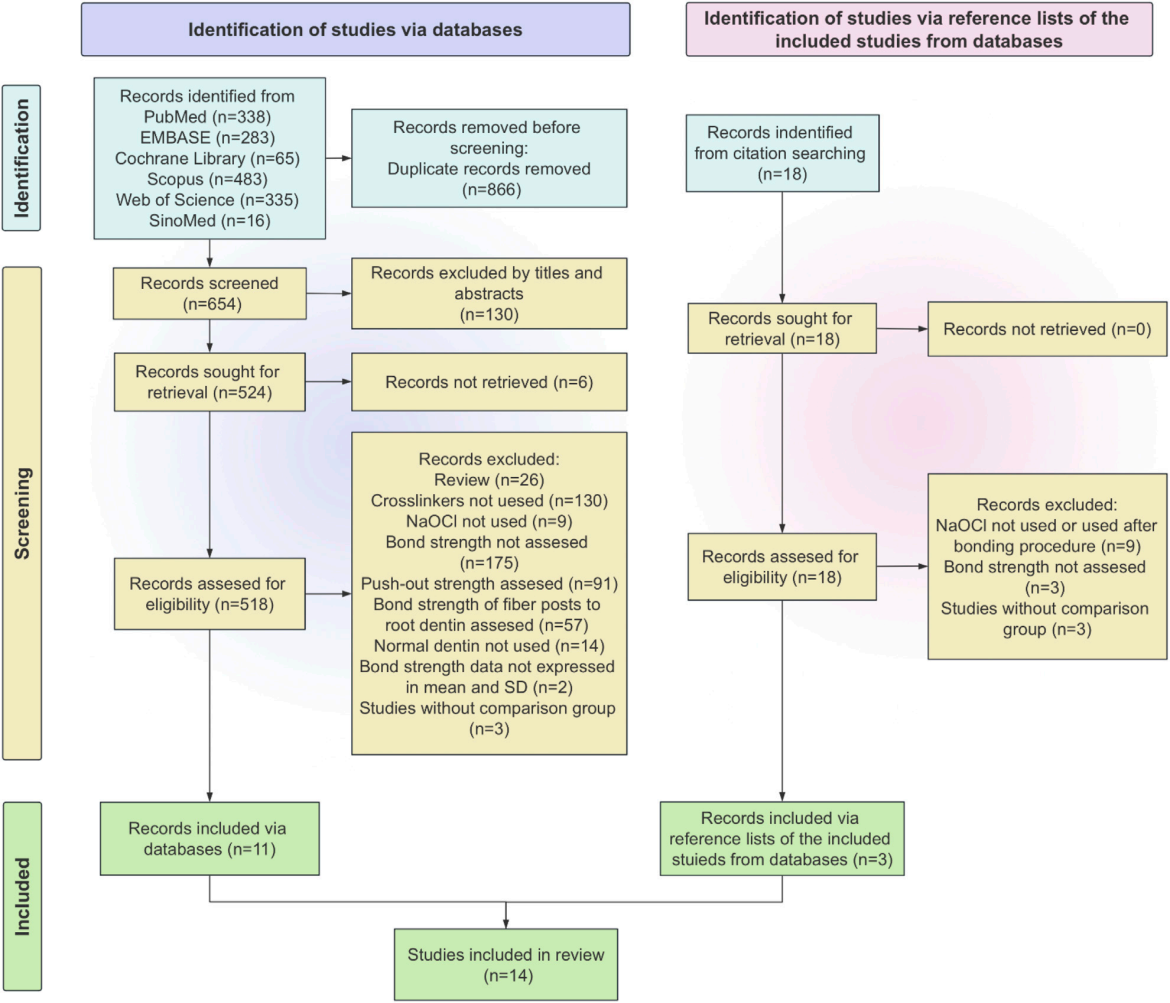
Flowchart of studies selection.

The exclusion criteria were: (1) reviews; (2) case reports; (3) incomplete data reported (results presented solely in figures without specifying the exact mean values and standard deviations); (4) lacking comparison group. Details of the studies selection and elimination are shown in Figure 1.

### 2.3 Data extraction

Duplicate records were identified using NoteExpress software. Subsequently, these duplicates were cross - checked by the authors (ZW and FS) and then eliminated. Two reviewers (ZW and FS) carried out the data extraction independently. If there were any questions, the study authors were contacted. Inter-rater reliability testing was implemented to assess the consistency of reviewers’ work. Conflicts were settled by consulting a third author (ZX). Relevant data were extracted and tabulated with Microsoft Office Excel. The following factors were taken into account when creating data extraction tables: author, year, tooth type, NaOCl concentration, NaOCl treated time, crosslinking agents, crosslinking agents’ concentration, adhesive used, bond strength test, predominant failure mode and main results (Table 2).

**TABLE 2:** Characteristics of the studies included in the review.

Author, year	Tooth type	NaOCl concen-tration	NaOCl treated time	Cross-linking agents	Crosslink-ing agents’ concen-tration	Collagen treatment time	Adhesive used	Bond Strength test	Major failure mode	Main results
Arslan, 2019 (Arslan et al., 2019)	Human molars 3 mm below the occlusal surface	2.5%	Not mentioned	EDTA	17%	Not mentioned	Clearfil SE Bond (Kuraray, Osaka, Japan)	SBS	Adhensive	There is no statistically significant difference in bond strength of NaOCl-treated dentin between EDTA group and control group
Barutcigil, 2014 (Barutcigil et al., 2014)	Human molars; pulp chamber roof	5%	5 min	EDTA	17%	5 min	Adper Scotchbond Multi-purpose (3M ESPE, St Paul, MN, United States); Adper SE Plus (3M ESPE); Clearfil S3 Bond (Kuraray Medical, Okayama, Japan); Silorane Bond (3M ESPE)	μTBS	Adhensive	NaOCl significantly reduced the bond strengths of all adhesives. The EDTA and NaOCl combination did not show a statistically significant reduction in bond strengths of the adhesives to pulpal dentin
Cecchin, 2010 (Cecchin et al., 2010)	Human molars 3 mm below the occlusal surface	1%	1 h	EDTA	17%	5 min	XENO III self-etching adhesive (Dentsply/DeTrey; Konstanz, Germany)	μTBS	—	The use of 1% NaOCl alone resulted in higher bond strength than the other treatments. The combination of 1% NaOCl and 17% EDTA produced similar bond strength to that of untreated dentin
Dikmen, 2015 (Dikmen, 2015)	Human molars 3 mm below the occlusal surface	5.25%	30 s	PA	5%	10 min	Single Bond Universal; Adhesive (3M ESPE; St Paul, MN, United States)	μTBS	Adhesive/Mix	The application of PA to NaOCl-treated dentin significantly improved the microtensile bond strength
Dikmen, 2018 (Dikmen and Tarim, 2018)	Human molars 3 mm below the occlusal surface	5.25%	30 s	EDTA SA	17% EDTA 10% SA	1 min 10 min	Adper Single Bond 2 (3M ESPE, St. Paul, MN, United States; Clearfil SE Bond (Kuraray Medical, Tokyo, Japan); Xeno 3 (Dentsply, DeTrey, Konstanz, Germany)	μTBS	Adhesive/Mix	Sodium ascorbate after NaOCl could restore compromised bond strengths
Farina, 2011 (Farina et al., 2011)	Human molars 3 mm below the occlusal surface	1%	40 min	EDTA	17%	5 min	Clearfil SE Bond self-etching adhesive (Kuraray; Okayama, Japan)	μTBS	Adhesive and mixed	The application of 2% CX followed by the application of 17% EDTA resulted in increasing the bond strength of the self-etching adhesive system to dentine, when compared with the results obtained for the other tested groups
Fawzi, 2010 (Fawzi, 2010)	Human molars; pulp chamber dentin flat	5.25%	10 min	EDTA	17%	1 min	Clearfil S3 Bond (Kuraray; Osaka, Japan); Adper Single Bond 2 (3M ESPE; St Paul, MN, United States)	SBS	—	The irrigant regimens examined could be used safely prior to bonding except for the NaOCl, which should be negated if it is to be followed by Adper Single Bond 2, and the etching step cannot be omitted if an etch-and-rinse adhesive system is the adhesive of choice
Kasraei, 2013 (Kasraei et al., 2013)	Human premolars 5 mm below the occlusal surface	2.5%	30 s	EDTA	0.5-M	30 s	I-Bond (heraeus Kulzer, hanau, Germany); Clearfil S3 Bond (Kuraray Co, Okayama, Japan)	μTBS	—	Application of EDTA or EDTA + NaOCl before one-step self-etch adhesives increased μTBS
Mohannad 2020 (Mohannad et al., 2020)	Human molars Not mentioned	5%	5 min	IP6 and EDTA	1%I P6 17% EDTA	IP6 1 min and 30 s EDTA 1 min	Scotchbond Universal adhesive (3M ESPE, St. Paul, MN, United States)	μTBS	Resin tags formed inside most of the dentinal tubules	IP6 reversed the adverse effects of NaOCl on resin-dentin adhesion without the chlorine-depleting effect of EDTA
Prasansuttiporn, 2017 (Prasansuttiporn et al., 2017)	Human molars; pulp chamber dentin flat	Not mentioned	30 s	RA	100 μM	5s	Clearfil SE Bond (Kuraray Noritake; Tokyo, Japan)	μTBS	Mixed Failure	Rosmarinic acid restored the compromised initial bond strengths to smear-layer–deproteinized dentin
Prasansuttiporn 2011 (Prasansuttiporn et al., 2011)	Human molars; flat surfaces under the sound dentin	6%	30 s	SA RA	10% SA 100 μM RA	5 s 10 s	Clearfil Protect Bond (Kuraray Medical Inc., Tokyo, Japan)	μTBS	Mixed Failure	The application of sodium ascorbate solution for 5 or 10 s did not significantly increase the compromised bonding to NaOCl-treated dentin Applying rosmarinic acid for 5 or 10 s improved bond strengths to NaOCl-treated dentin
Santos, 2006 (Santos et al., 2006)	Bovine incisors pulp chamber dentin flat	5.25%	30 min	EDTA	17%	5 min	Clearfil SE Bond (Kuraray, Kurashiki, Japan)	μTBS	Mixed and interfacial	There was a significant decrease in bond strength associated to NaOCl, whereas chlorhexidine irrigation showed no effects on adhesion
Vongpha, 2005 (Vongphan et al., 2005)	Human molars; 3 mm below the occlusal surface	5.25%	10 min	SA	10$	10 min	Single bond (3M-ESPE, St Paul, MN, United States)	μTBS	Cohensive	Sodium hypochlorite significantly reduced the bond strengths of the adhesive when a total-etching was applied. The application of sodium ascorbate on sodium hypochlorite treated dentine significantly
Wang, 2019 (Wang et al., 2019)	Human molars Flat surfaces of sound dentin in the middle of the tooth	5.25%	20 min	PA	5% 10% 15%	1 min 5 min 10 min	Clearfil SE Bond (Kuraray Medical Inc., Tokyo, Japan)	μTBS	Adhesive and Mixed	Microtensile bond strength to NaOCl-treated dentine recovered after the application of either 5% PA for more than 5 min or 10% or 15% PA for more than 1 min. The application of PA before an adhesive procedure may immediately restore the compromised bond strength of NaOCl-treated dentine

SBS, shear bond strength test; μTBS, micro-tensile bond strength test.

### 2.4 Quality assessment

Based on and modified from previous study, (Hardan et al., 2022), risk of bias was assessed by two reviewers according to the following parameters: standardized specimens, specimen randomization, teeth free of caries/restoration, sample size calculation, blinding of sampling and assessment, manufacturer’s instructions, control groups, failure mode and incomplete outcome data. If the parameter was included or carried out correctly, the study received a “Y,” but if it was absent or carried out insufficiently, it received a “N.” As shown in Table 3, of the characteristics that scored “Y,” 1 to 3 denoted a high risk of bias, 4 to 6 a medium risk, and 7 to 9 a low risk. Interviewing the third reviewer (ZX) settled any disputes that arose between the two investigators. Inter-rater reliability testing was implemented to assess the consistency of reviewers’ assessment.

**TABLE 3:** Risk of bias of the included studies.

Author year	Standardized specimens	Specimen randomization	Teeth free of caries/restoration	Sample size calculation	Blinding of sampling and assessment	Manufacturer ‘s instructions	Control groups	Failure mode	Incomplete outcome data	Risk of bias
Arslan 2019	Y	Y	Y	N	N	Y	Y	Y	Y	low
Barutcigil 2014	Y	Y	Y	N	N	Y	Y	Y	Y	low
Cecchin 2010	Y	Y	Y	N	N	Y	Y	N	Y	medium
Dikmen 2015	Y	Y	Y	N	N	Y	Y	Y	Y	low
Dikmen 2018	Y	Y	Y	N	Y	Y	Y	Y	Y	low
Farina 2011	Y	Y	Y	N	N	Y	Y	Y	Y	low
Fawzi 2010	Y	Y	Y	N	N	Y	Y	N	Y	medium
Kasarei 2013	Y	Y	Y	N	N	Y	Y	N	Y	medium
Mohannad 2020	Y	Y	Y	N	N	Y	Y	Y	Y	low
Santos 2006	Y	Y	Y	N	N	Y	Y	Y	Y	low
Prasansuttiporn 2011	Y	Y	Y	N	N	Y	Y	Y	Y	low
Prasansuttiporn 2017	Y	Y	Y	N	N	Y	Y	Y	Y	low
Vongphan 2005	Y	Y	Y	N	N	Y	Y	Y	Y	low
Wang 2019	Y	Y	Y	N	N	Y	Y	Y	Y	low

### 2.5 Meta-analysis

The meta-analysis was performed separately for each crosslinking agents using RevMan 5.3 software. Subgroup comparisons were performed based on the duration of NaOCl exposure. Studies of EDTA group were analyzed separately according to the concentration of NaOCl (NaOCl greater than 2.5% or less than 2.5%). The level of significance for all tests was 5%. Statistical heterogeneity among studies was measured by the Chi^2^ test and the I^2^ test, with values greater than 50% were considered to indicate of substantial heterogeneity (Higgins, 2003). Depending on the value of heterogeneity, the meta-analysis used standardized mean difference (SMD), random effects model or fixed effects model. Leave-one-out sensitivity analysis was used to evaluate the reliability of the meta - analysis results (Deeks et al., 2008).

## 3 Results

From 1,538 potentially eligible studies obtained from six databases, 14 studies were selected for full-text analysis. Figure 1 is a flowchart that summarizes the studies selection process according to the PRISMA Statement. Characteristics of included studies.

All the studies were in English. EDTA (Santos et al., 2006; Cecchin et al., 2010; Fawzi, 2010; Farina et al., 2011; Kasraei et al., 2013; Barutcigil et al., 2014; Dikmen and Tarim, 2018; Arslan et al., 2019; Mohannad et al., 2020) was the most used crosslinking agent for nine studies. Three studies were conducted to investigate SA, (Vongphan et al., 2005; Prasansuttiporn et al., 2011; Dikmen and Tarim, 2018), while two studies were conducted for each of PA (Dikmen, 2015; Wang et al., 2019) and RA (Prasansuttiporn et al., 2011; Prasansuttiporn et al., 2017). A single study was conducted on IP6 (Mohannad et al., 2020). The characteristics of included studies are shown in Table 2. The results of the inter-reviewer agreement test revealed a high level of consistency (Kappa value = 0.883, CI 0.786–0.961), suggesting that the results are relatively reliable.

### 3.1 Meta-analysis

**FIGURE 2:**
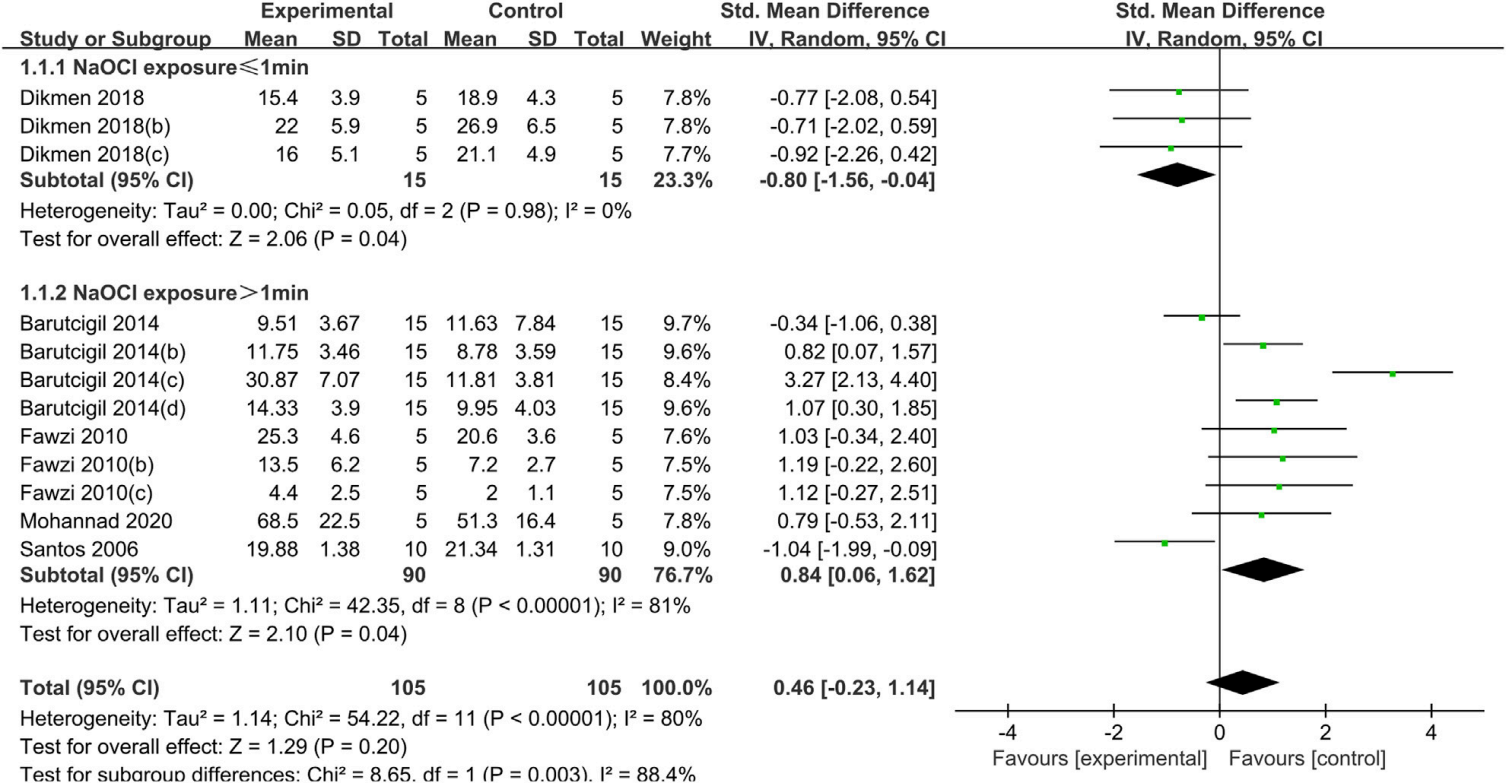
Forest plot of the BS comparison between the EDTA treated group and the control group according to the high concentration NaOCl exposure time.

**FIGURE 3:**
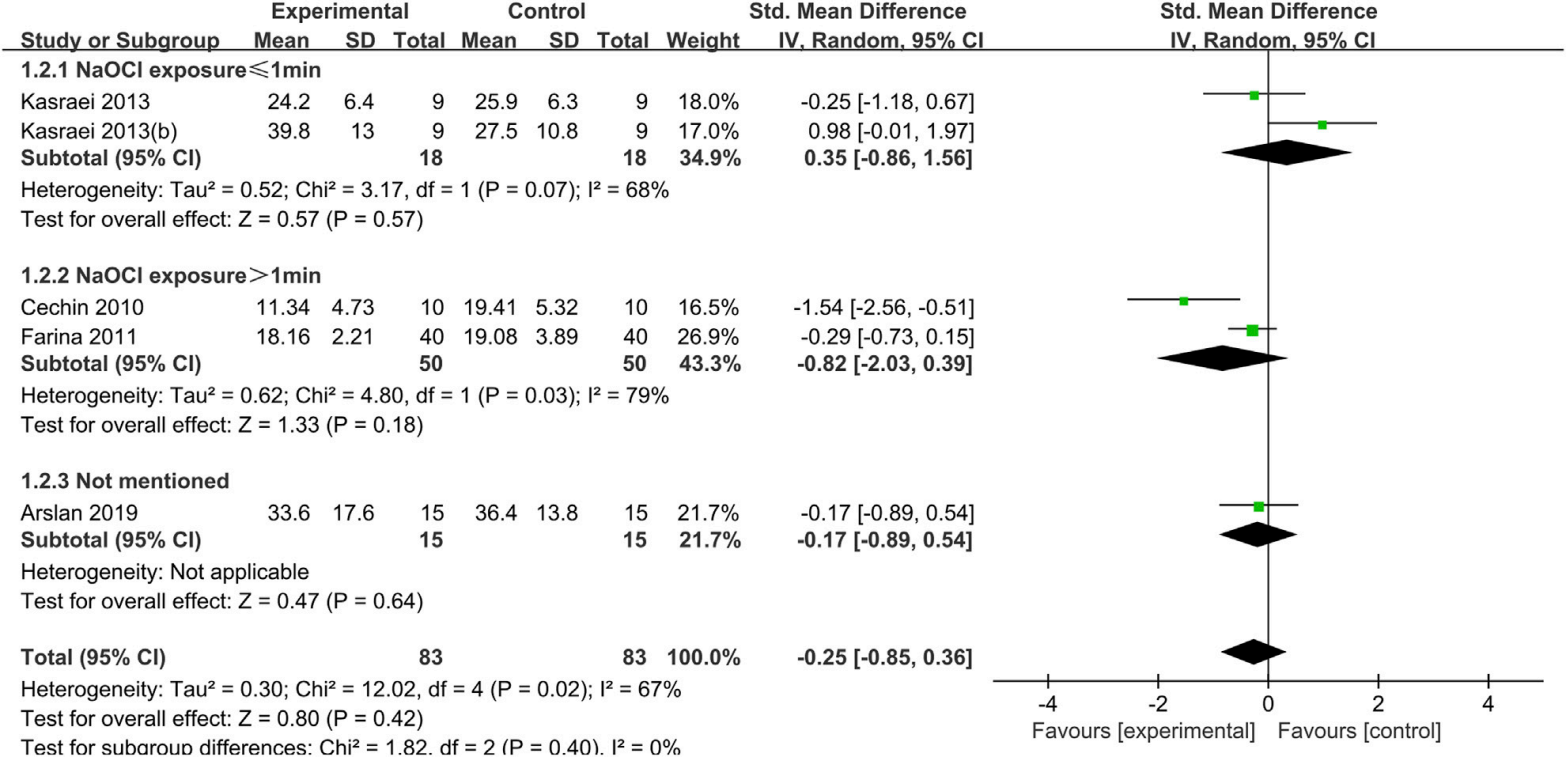
Forest plot of the BS comparison between the EDTA treated group and the control group according to the low concentration NaOCl exposure time.

**FIGURE 4:**
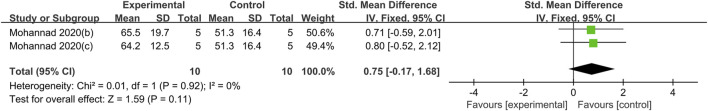
Forest plot of the BS comparison between the IP6 treated group and the control group.

**FIGURE 5:**
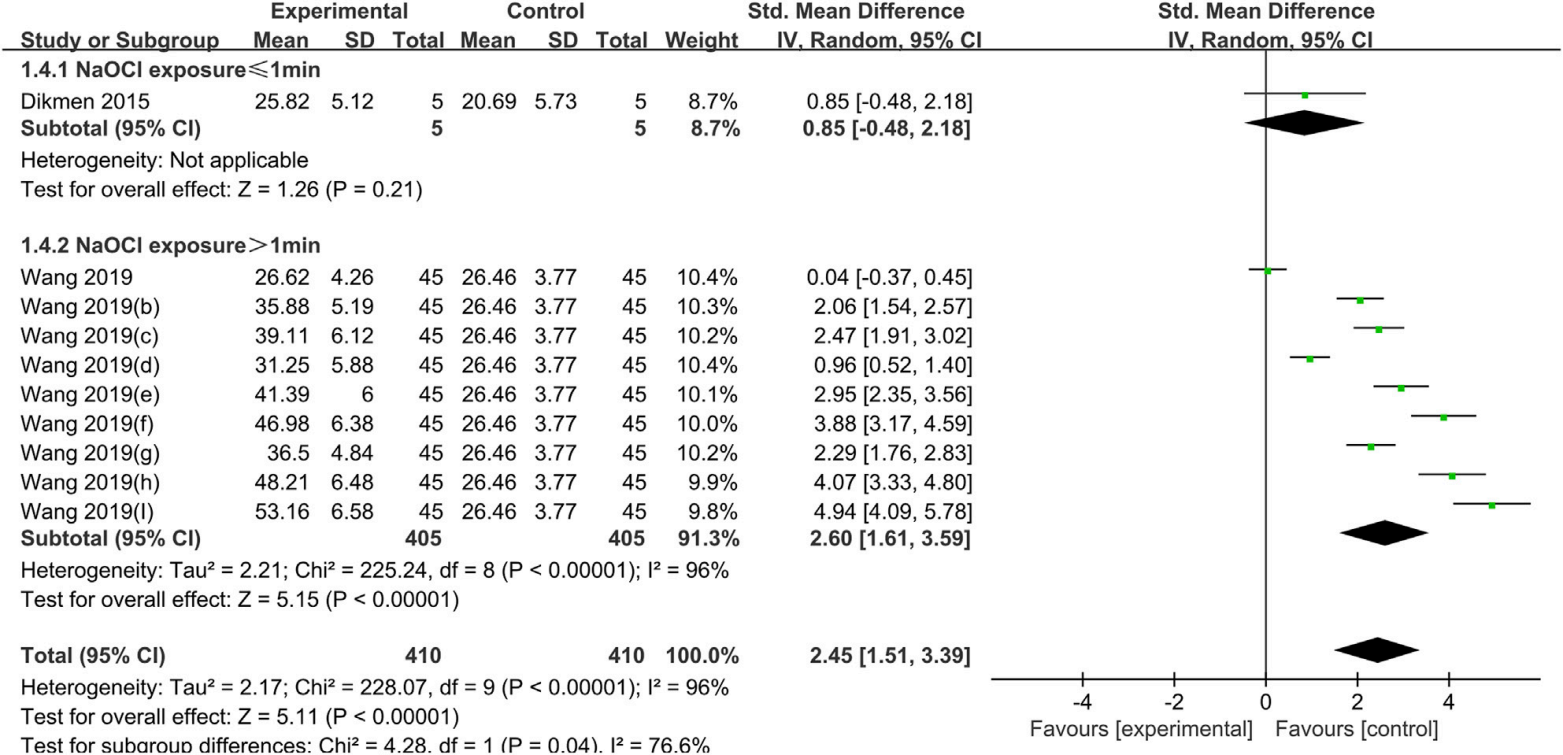
Forest plot of the BS comparison between the PA treated group and the control group according to the high concentration NaOCl exposure time.

**FIGURE 6:**
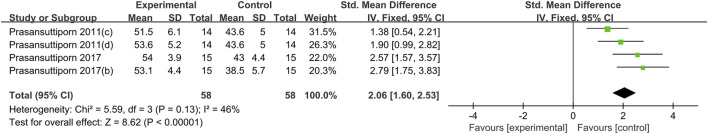
Forest plot of the BS comparison between the RA treated group and the control group.

**FIGURE 7:**
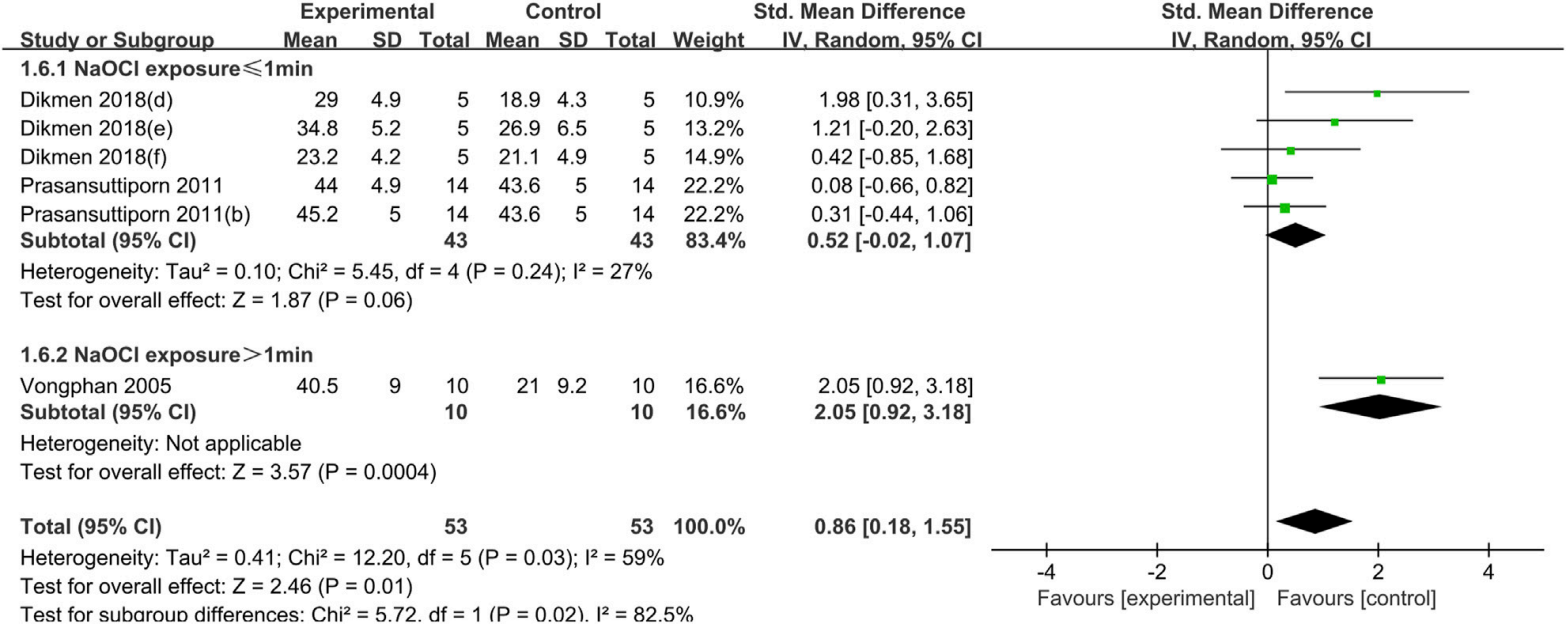
Forest plot of the BS comparison between the SA treated group and the control group according to the high concentration NaOCl exposure time.

This review incorporates several substances that have been identified as crosslinking agents, including EDTA, IP6, PA, RA and SA. The crosslinking agents were applied to dentin after NaOCl for irrigation and prior to composite resin bonding. Different crosslinkers used as a pretreatment were analyzed separately. Furthermore, separate analysis of EDTA group were conducted for the concentration of NaOCl (NaOCl greater than 2.5% or less than 2.5%). Given the relatively high heterogeneity of the meta-analysis, after heterogeneity analysis and treatment, the data of EDTA, PA and SA meta-analysis were divided into two subgroups according to the duration of NaOCl exposure and analyzed using a random effects model. The results of meta-analysis are presented in the following Figures 2–7.

#### 3.1.1 EDTA

The use of EDTA was statistically significant in weakening the bond strength of dentin to composite resin when dentin was exposed to a high concentration of NaOCl solution (concentration greater than 2.5%) for a short period of time (less than or equal to 1 min) (SMD:-0.80; CI:-1.56,-0.04; *P* = 0.04). Conversely, when dentin was exposed to high concentrations of NaOCl for more than 1 min, the use of EDTA had a statistically significant effect on enhancing the bond strength (SMD: 0.84; CI:0.06,1.62; *P* = 0.04). (Figure 2).

When dentin was exposed to low concentrations of NaOCl (concentrations less than or equal to 2.5%), the difference between the bond strength of dentin treated with EDTA and that of the control group was not statistically significant, regardless of the duration of exposure (Figure 3).

#### 3.1.2 IP6

Two comparisons in Mohannand 2020, (Mohannad et al., 2020), was included in the meta-analysis of IP6. The concentration of NaOCl used in this study was 5% with a 5-min exposure. Mohanna, 2020 (b) was treated with IP6 for 1 min and Mohannad 2020 (c) was treated 30 s. The meta-analysis demonstrated that IP6 application on NaOCl-treated dentin BS exhibited no statistically significant differences (SMD: 0.75; CI: −0.17, 1.68; *P* = 0.11) (Figure 4).

#### 3.1.3 PA

NaOCl solution at a concentration of 5.25% was used in all studies crosslinked with PA, and the difference in dentin bond strength between the PA experimental group and the control group was not statistically significant for exposure times of less than 1 min (SMD:0.86; CI: −0.85,2.18; *P* = 0.21); for exposure times of greater than 1 min, PA significantly increased bond strength (SMD: 2.60; CI:1.61,3.59; *P* < 0.001) (Figure 5).

#### 3.1.4 RA

NaOCl exposure time for each of the four comparisons using PA crosslinking was 30 s. The meta-analysis revealed that RA significantly increased the BS between NaOCl treated dentin and composite resin (SMD: 2.06; CI: 1.60, 2.53; *P* < 0.001) (Figure 6).

#### 3.1.5 SA

The use of NaOCl at concentrations above or equal to 5.25% was a common feature of both studies utilizing SA cross-linking. The results demonstrated that SA exerted a weak effect on the bond strength of dentin when the exposure time was less than 1 min (SMD: 0.52; CI: −0.02, 1.07; *P* = 0.06). However, this difference was not statistically significant. When the exposure time to sodium hypochlorite was extended, a significant increase in the bond strength of dentin was observed in the presence of SA (SMD:2.05; CI:0.92, 3.18; *P* < 0.001) (Figure 7).

### 3.2 Sensitivity analysis

A meta-analysis of studies utilizing different crosslinking agents were conducted separately, with leave- one-out sensitivity analyses performed. No significant alterations in the results were observed upon the exclusion of any of the articles, and the results of the meta-analysis were deemed to be robust.

### 3.3 Risk of bias

**FIGURE 8:**
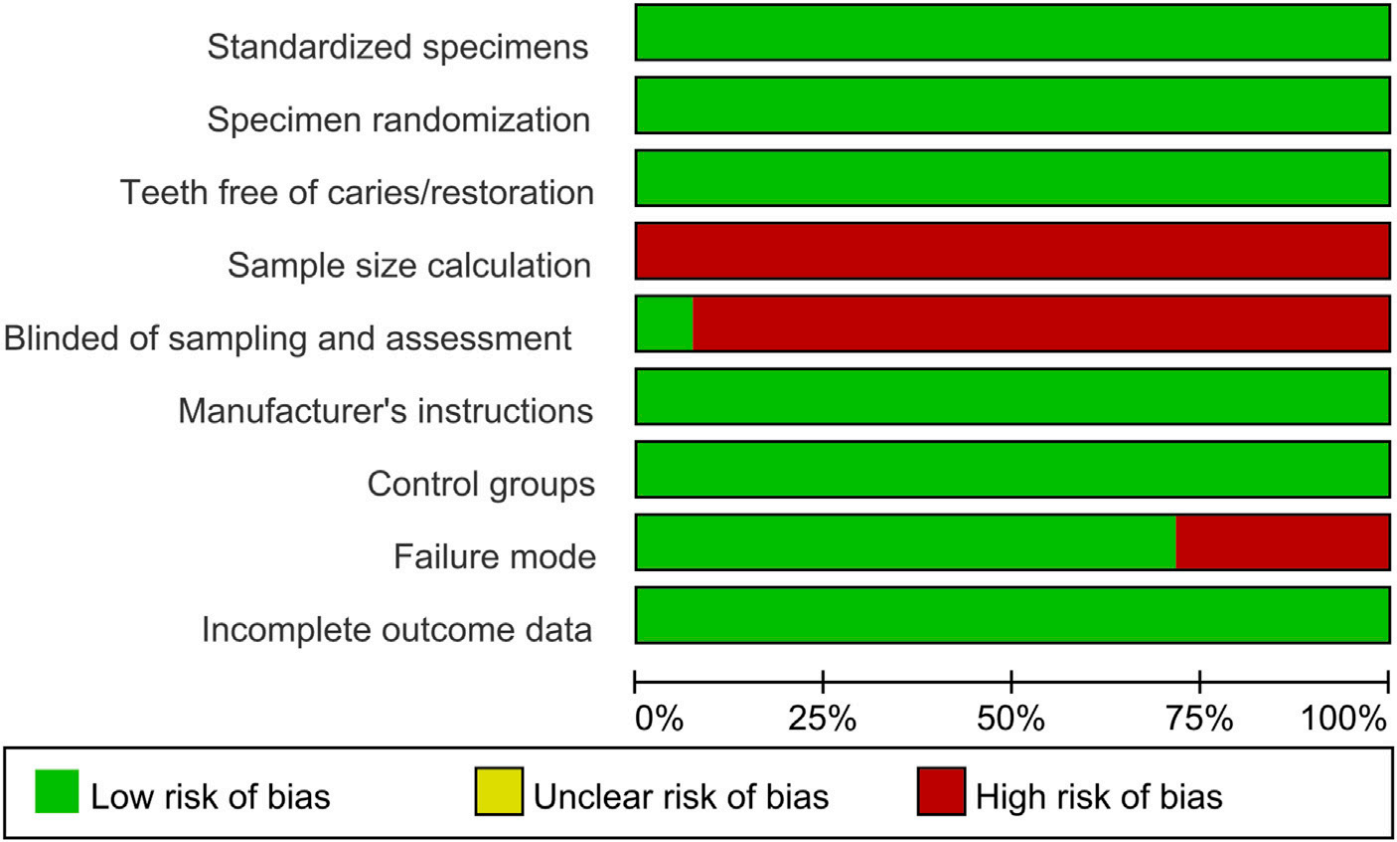
Summary of risk of bias.

Most of the studies were categorized as having low risk of bias based on the standards set forth for the risk of bias assessment. Regarding the aforementioned criteria, approximately 25% of the studies did not report information about failure mode, the majority did not report blinding of sampling and assessment, and all did not perform sample size calculations (Figure 8). The result of the inter - reviewer agreement test manifested a high degree of consistency. The Kappa value was 0.897, with a 95% confidence interval ranging from 0.806 to 0.971, which implies that the results are relatively dependable.

## 4 Discussion

This is the first systematic review and meta-analysis to investigate the relationship between the BS of NaOCl-treated dentin and three variables: the cross-linker used, the concentration of NaOCl and the time of NaOCl exposure. The results of the meta-analysis indicated that the effect of collagen crosslinkers on the BS of NaOCl treated dentin was influenced by the concentration of NaOCl and the duration of exposure. When the NaOCl exposure time was extended to more than 1 min with concentrations greater than 2.5%, collagen crosslinkers including EDTA, PA and SA all demonstrated a significant positive effect on bond strength. Consequently, the hypothesis put forth in this review was found to be partially rejected.

EDTA, PA and SA are available for comparison between different exposure durations at high NaOCl concentration. The results showed that when higher concentrations of NaOCl were used and the exposure time was less than 1 min, none of the EDTA, PA, and SA had a statistically significant effect on dentin BS compared to the control group. When the exposure time was extended to more than 1 min with high concentrations of NaOCl, EDTA, PA and SA all demonstrated a significant positive effect on bond strength. It can be inferred that the effect of EDTA, PA and SA on the BS of NaOCl treated dentin was influenced by the concentration of NaOCl and the duration of exposure. This may be since the negative effect of NaOCl on dentin is only gradually apparent when higher concentrations act for a longer period. Taniguchi et al. (2009) treated dentin with 6% NaOCl for 15 s and did not observe a significant change in dentin adhesive strength, whereas in the case of Kambara et al. (2012), there was even a significant increase in dentin bond strength after treatment with 1% sodium hypochlorite solution for 15 s. However, dentin bond strengths treated with 2.5% and 5.25% NaOCl for 20 min were significantly lower than those of the negative control group, with a decrease of 46% and 50.2%, respectively (Wang and Liang, 2017).

Only the studies using EDTA crosslinking had an experimental group using a low concentration of sodium hypochlorite, and the studies using the other crosslinking agents used a high concentration of NaOCl. The meta-analysis showed that regardless of whether the duration of exposure of dentin to a low concentration of sodium hypochlorite was 1 h, 40 min, 30 s, or some other unmentioned period (Arslan et al., 2019), the application of EDTA had no statistically significant effect on BS. This may also be due to the fact that at lower concentrations, NaOCl does not cause significant changes in BS. Cecchin et al. (2010) treated dentin with 1% NaOCl during 1 h (reapplied every 5 min) and found that it lead to a higher BS of Xeno III system to dentin, they explain this outcome may be concerned with the superficial morphology of dentin treated with NaOCl was not significantly changed for that 1%NaOCl does not remove the smear layer and expose the dentinal tubules (Garberoglio and Becce, 1994).

### 4.1 EDTA & IP6

NaOCl and EDTA are the two most used irrigants in endodontic treatment. EDTA is an effective metal chelating compound with a high antioxidant potential (Let et al., 2007; Thbayh et al., 2023). It has been suggested that dentin treated with EDTA exhibits an increased resistance to degradation by NaOCl at the resin-dentin interface (Torii et al., 2003; Kim et al., 2011). Furthermore, EDTA exerts an inhibitory effect on matrix metalloproteinase (MMP), which contributes to the durability of resin-dentin bonding. Nevertheless, this effect is time-limited (Thompson et al., 2012; Toledano et al., 2012). EDTA is a less hazardous and more affordable cross-linking agent than traditional cross-linkers like epichlorohydrin (EPI) and 1-ethyl-3-(3-dimethylaminopropyl)-carbodiimide hydrochloride (EDC) (Zhao et al., 2017). From results above, it can be observed that the effect of EDTA on the BS is related to the exposure time of NaOCl at different concentrations. The interaction between the two may be related to the following reasons: First, the interaction of pH. Weak acidity of EDTA (pH 7.2) may neutralize the alkalinity of high-concentration NaOCl, reducing the effective chlorine concentration and weakening its antibacterial ability. However, this pH change has a relatively small impact during short - term exposure, while it may be more significant during long-term treatment. Second, high-concentration NaOCl rapidly dissolves the surface collagen, but does not completely remove the deeper layer organic residues within a short time. At this time, the demineralization effect of EDTA exposes more unmineralized collagen fibers, but these fibers are structurally loose due to the oxidative damage of NaOCl and cannot form an effective hybrid layer. Meanwhile, excessive demineralization leads to a decrease in the micro-hardness of the dentin surface, weakening the mechanical interlocking effect (Barón et al., 2013). Third, relatively long-time NaOCl exposure treatment thoroughly removes organic residues and the smear layer, forming a clean inorganic surface. EDTA further removes the hydroxyapatite debris after demineralization, opening the dentinal tubules, increasing the surface area and the resin penetration depth (De-Deus et al., 2006). Therefore, combined with the pretreatment of high concentration NaOCl for a relatively long time, the micromechanical retention between the resin and dentin is significantly enhanced.

IP6 is the primary phosphorus storage type found in plant seeds and bran (Raboy, 2003). It is extracted from natural plants that include six phosphate groups, making it a crosslinking agent that offers a large number of feasible crosslinking sites to help in the construction of three-dimensional macrostructures (Liu et al., 2024). In specific circumstances, IP6 can help form a ternary complex (Protein-CaPhytate) that has good chemical bond (Cheryan, 1980) and has been used in the construction of conductive hydrogels (Zhang et al., 2019; Liu et al., 2024). Similar to EDTA, with its remarkable capacity to chelate with multivalent cations including calcium, magnesium, and iron, IP6 is a highly negatively charged molecule (Luttrell, 1993; Torres et al., 2005). It functions ais an antioxidant, resisting oxidative reactions through the trapping of free radicals, reduction of their production and scavenging. Dentin surfaces treated with IP6 were free of smear layer and smear plugs, dentinal tubules opened and intertubular collagen was visible (Souparnika et al., 2023). IP6 has been identified as a potential etchant and chelating agent, given its acidic and chelating properties. It was reported can be used as a substitute for phosphoric acid and EDTA in clinical practice (Nassar et al., 2015; Kong et al., 2017; Gandhi et al., 2020).

In addition to considering the concentration of sodium hypochlorite and the duration, the fact that EDTA and IP6 not only function as antioxidants and crosslinking agents but also participate in the chemical reactions preceding bonding in the capacity of chelating agents. This may explain the outcome that EDTA declined the BS of NaOCl treated dentin in certain situation as well as IP6 exerts not significant positive effect on BS. Recognized as the most efficacious chelating agent with excellent lubricity, EDTA is commonly used in endodontic therapy (Calt and Serper, 2002; Barutcigil et al., 2014). It was reported that the demineralizing effect is pronounced, with the potential for dentin softening, dentinal tubules enlargement and collagen fibrils denaturation (Calt and Serper, 2002). Formation of the hybrid layer and the durability of bonding quality are consequently affected. Compare with EDTA, the application of IP6 for an equivalent period of time results in the removal of the smear layer in a more effective manner. According to Souparnmika et al., it was observed that although EDTA resulted in a nearly clean dentinal surface with open dentinal tubules while IP6 produced a dentinal surface free of debris with a higher number of open tubule, which both (Souparnika et al., 2023).

Although a notable elevation in BS was not observed in the meta-analysis of IP6, the BS of the experimental groups to which the IP6 treatment was applied was higher than that of the control group in all cases. This contrasts with the findings that EDTA exerts both beneficial and detrimental effects on BS. This may be explained by the fact that 1% IP6 (pH 1.3) has a higher acidity than 17% EDTA (pH 7.2), thereby has a superior capacity to neutralize the residual NaOCl on the dentin surface. Furthermore, Mohannad et al. (2020) reported that IP6 exhibited superior biocompatibility and smear layer removal capabilities on flat dentin surfaces in comparison to EDTA. Additionally, IP6 demonstrated better dentin tubules opening, which may facilitate enhanced penetration of the resin protrusion into the dentin tubules of the root canal. Moreover, IP6 had a noticeably stronger inhibitory effect on collagen breakdown than did PA and EDTA (Kong et al., 2017).

Based on the results of meta-analysis of the articles included in this study, it was concluded that the effect of IP6 on the BS of NaOCl-treated detin is not statistically significant. Despite the considerable efforts of many scholars to explore the use of IP6 in dentin bonding, (Muana et al., 2019; Yadav et al., 2021; Attia et al., 2022; Souparnika et al., 2023), here remains a paucity of research exploring the impact of IP6 on BS of NaOCl-treated dentin. Although our study provides some evidence for the effectiveness of IP6, current study has limitations in fully validating the effectiveness of IP6 as a crosslinking agent and that more studies are needed to provide robust evidence. Further validation is necessary before it can be widely recommended for use in clinical.

### 4.2 PA & RA

PA, RA are crosslinkers with excellent antioxidant properties (Huerta-Madroñal et al., 2021; Guan et al., 2022; Noor et al., 2022). In addition, they have been demonstrated to process the ability to inhibit MMP (Epasinghe et al., 2013). PA are natural crosslinkers extracted from polyphenolic compounds (Rauf et al., 2019). Regarded as one of the most active dentin tissue biomodifiers used in the dental bonding procedure, (Alkhazaleh et al., 2022), PA have the potential to enhance the mechanical properties of dentin, thereby improving the quality of the hybrid layer (Anovazzi et al., 2024).

The degree of heterogeneity observed in the meta-analysis of PA was considerable (I^2^ = 96%), as illustrated in Figure 5. The source of the heterogeneity may be attributed to the three concentrations of 5%, 10%, and 15% and the three durations of PA treatment (1 min, 5 min, and 15 min, respectively) used in the study by Wang et al. (2019) Higher concentrations of PA can form a denser collagen matrix, which can impede the water leaching and reduce the vapor permeability of the proanthocyanidin–collagen film (Balalaie et al., 2018). It is therefore appropriate to conclude that the concentration and treatment time are also important for PA to reverse the adverse effect of NaOCl on dentin BS. Higher PA concentrations and longer durations will result in greater BS recovery.

Similar to PA, RA is a polyphenolic flavonoid extracted from rosemary, which has crosslinking and MMP-inhibitory abilities, as well as a high antioxidant capacity (Hawkins and Davies, 1999; Apak et al., 2008). The treatment times in the studies (Prasansuttiporn et al., 2011; Prasansuttiporn et al., 2017) were all within the interval of 5–10 s, which is the shortest for rosemarinic acid compared to other cross-linking agents. However, the results of the meta-analysis indicate that a statistically significant increase in the BS of NaOCl-treated dentin could still be observed after treatment with RA, suggesting that RA enhances the BS of NaOCl-treated dentin with a shorter treatment time. The efficient reversal of the reduction in BS of NaOCl-treated dentin by rosemarinic acid may be attributed to the fact that RA contains p-toluenesulfinic acid sodium salt. P-toluenesulfinic acid sodium salt is present in a product known as Accel (Sun Medical Co. Ltd.), which is used commercially as a pretreatment agent for adhesive root canal sealers to lessen or completely eliminate the oxidative effect of NaOCl (Khoroushi and Kachuei, 2014). In additionr, p-toluenesulfinic acid sodium salt can accelerate polymerization of composite resin (Apak et al., 2008). The polymerization of composite resin can be accelerated by the sodium salt of p-toluenesulfinic acid (Bowen, 1965; Taniguchi et al., 2009).

In addition, the solvent for RA was 5% ethanol in the two included studies that used RA as a cross-linking agent, and aqueous solutions of RA at different concentrations were used as cross-linking agents in other studies on RA. As a natural extract, the physiological activity of PA is influenced by a variety of factors including the extraction process and the solvent (Han et al., 2003; Ku et al., 2007). Aydi et al. (2016) found that the solubility of RA decreases with an increase in water content. Previous studies (Liu et al., 2011; Fang et al., 2012) found that the ultimate tensile strength of collagen in the PA-treated group was higher than that in the water-solvent group for the same treatment time in the ethanol-solvent group and the acetone-solvent group, suggesting that ethanol may be a more suitable solvent for PA pretreatment. This may be associated with the hydrogen bonding capacity of these solvents, as gauged by the Hansen solubility parameter for hydrogen bonding, δH (Chappelow et al., 2000). The δH values of ethanol and acetone were found to be lower than that of distilled water, indicating that the weaker bond-forming solvent would occupy fewer hydrogen bonding sites. The additional available hydrogen bonding sites, in conjunction with the collagen structure, may facilitate the formation of new hydrogen bonds between PA collagen or collagen-collagen molecules, thereby inducing collagen cross-linking and resulting in enhanced mechanical properties (Nalla et al., 2005). Furthermore, Hagerman and Klucher (1986) have demonstrated that ethanol, rather than acetone, can reduce the dielectric constant of the medium and stimulate PA-collagen.

### 4.3 SA

The SA functions as a reducing agent, facilitating the interaction between oxygen and the NaOCl by-product (Vongphan et al., 2005). It has been reported that SA can reverse the negative effects of NaOCl on the polymerization of dentin bonding agent (Morris et al., 2001). Furthermore, by serving as an electron donor and scavenging free radicals, it can restore the BS (Weston et al., 2007) and permit full polymerization without prematurely stopping the process (Lai et al., 2001). Additionally, SA eliminates the vertical shag-carpet-like nanoleakage pattern created by NaOCl due to incomplete penetration of resin into demineralized dentin (Kewlani et al., 2020).

The studies using SA crosslinking employed high concentrations of NaOCl solutions. The subgroup with an exposure time of less than 1 min did not observe a significant increase in bond strength by SA. In addition to the previously mentioned reason for the short duration of the NaOCl application, the following factors may also be related to this outcome. In Prasansuttiporn, 2011 (a) (b), (Prasansuttiporn et al., 2011), 10% SA was applied for 5 and 10 s, which may not be sufficiently long to facilitate the formation of a strong bond between NaOCl-treated dentin and the self-etch adhesive agent. It is possible that an extended application period may have a significant impact on BS. Also, the location from which the dentin was sampled may be concerned. The majority of the studies included herein sampled the readily accessible extracted third molars for specimen preparation. The morphology and size of human third molars exhibit considerable variability, as do the shapes of their pulp chambers. Two principal methods are employed for the preparation of specimens. In Dikmen and Tarim (2018), for instance, the teeth were sectioned 3 mm below the occlusal surface, so do Kasarei et al. removed 5 mm of occlusal dentin to expose deeper superficial dentin without pulp exposure (Kasraei et al., 2013). An alternative approach was employed by Barutcigil et al. (2014) who sectioned the teeth through the pulp chamber. The orientation of dentin tubules is highly variable in different parts of the tooth (Liu et al., 2020). Some studies have shown that the structure of dentin tubules exerts a significant influence on the physical properties of dentin itself and their morphological distribution at the bonding interface affects the BS between the bonding system and dentin (Lin et al., 2017). The number of dentin tubules in the dentin on the side away (superficial dentin) from the pulp is fewer in number, with a correspondingly low density. The inter-dentin tubule matrix constitutes a large proportion of dentin tubules, and the distribution of collagen fibers is more concentrated than that observed in dentin on the side near the pulp (deeper dentin) (Guo et al., 2018). There are also more bonding sites with the resin bonding agent. Consequently, the resin adhesive has a higher penetration rate in the superficial dentin, which penetrates more rapidly than deeper dentin. In Dikmen’s experiments, dentin specimens were taken from relatively shallow dentin, and the thickness of the mixed layer at the interface is thinner than that of the corresponding total-etch bonding mixed layer. The quality of this hybrid layer is a critical determinant of BS (Perdigão et al., 2013; Frassetto et al., 2016).

### 4.4 Limitations and future direction

Researchers reported that the effect of NaOCl on dentin BS varies with chemistries of the bonding systems (Vargas et al., 1997; Prati et al., 1999; Frankenberger et al., 2000; Perdigão et al., 2000; Ishizuka et al., 2001; Stevens, 2014). As reported by Nikaido et al. (1999), the application of NaOCl in root canal treatment has been shown to have a detrimental effect on the adhesion of total-etch adhesive systems, with a comparatively minimal effect on self-etch primer systems. The technique sensitivity of total-etch adhesive system arises from the challenge of regulating the moisture content of etched dentin, which is essential to prevent collagen collapse and subsequent impediment of resin monomer infiltration (Spencer and Wang, 2002). The chemical products of the crosslinking agent and NaOCl can affect the dentin wetting of dentin prior to bonding, thus affecting the BS. This review did not impose restrictions on the types of adhesives in the included articles. Nevertheless, the majority of the studies included in this review used the Self-etch adhesive system, with only three studies using the total-etch adhesive system. Further studies using the total-etch adhesive system are required to elucidate the effect of crosslinking agents on the BS of NaOCl-treated dentin in the presence of different adhesive systems.

In addition, although the most commonly used clinical concentration of NaOCl is currently 5.25%, (Cai et al., 2023), and most of the NaOCl rinses used in the included articles were at a concentration of 5.25% and above, there is growing evidence that higher concentrations of NaOCl are more disruptive to the physicochemical properties and microstructure of dentin as well (Vargas et al., 1997; Boutsioukis and Arias-Moliz, 2022). This is evidenced by the fact that they are more likely to result in reduced BS as well as an elevated risk of root fracture (Erdemir et al., 2004; Pascon et al., 2009; Gu et al., 2017; Li et al., 2022; Xu et al., 2022). Some researchers have advocated the use of lower concentrations of NaOCl in clinical practice (Abuhaimed and Abou Neel, 2017; Boutsioukis and Arias-Moliz, 2022). However, it has also been suggested that irrigating with varying concentrations of NaOCl does not influence the compressive strength of dentin or the clinical outcome (Verma et al., 2019; Barakat et al., 2024). Further research into the effect of cross-linking agents on the BS of dentin treated with NaOCl at different concentrations is awaited.

It is also important to note that failure of a bonded restoration of a crown does not only occur in the immediate postoperative period. Rather, it is often observed after a certain period of use (Mokeem et al., 2023). The durability of hybrid layers has been demonstrated to be limited (Hashimoto et al., 2003; Pashley et al., 2004; Breschi et al., 2008). This outcome may be attributed to a multitude of factors, including inadequate resin monomer penetration into the demineralized dentin and unpolymerized monomer elution from the polymeric adhesive (Okuda et al., 2002; Breschi et al., 2008; Maravic et al., 2017). It is possible that these collagen fibers, which are exposed within the hybrid layer (incompletely infiltrated), may be affected by degradation. MMP are present in dentin in a dormant form and are triggered in low pH environments, which are similar to the conditions that occur during etching and caries processes (Mazzoni et al., 2015). Furthermore, all of the aforementioned changes require a certain amount of time to occur (Frassetto et al., 2016). However, with the exception of two of the included studies, which investigated long-term changes in BS, one of the limitations of the remaining studies was that they only evaluated the immediate recovery effect of crosslinking agents on BS to NaOCl-treated dentine. The durability of the improvement in bond strength of crosslinkers to NaOCl-treated dentin over time requires further investigation.

Finally, the studies included in this review generally lack the reporting of sample size calculation and the implementation of blinding. The calculation of sample size has a significant impact on the test power of a study and poses an obstacle to the assessment of heterogeneity. Inadequate implementation of blinding can lead to observational bias, affecting the generalizability of the meta-analysis results. This reflects the lack of experimental design in the research of this field or the omission of reporting such important information when writing articles. Therefore, special attention should be paid in future studies. In addition, all the included studies were all *in vitro* experiments using extracted human or bovine teeth, and the outcomes explored were limited to BS. *In vivo* studies are scarce. There is a paucity of observations on the effects of crosslinking agents on microleakage, secondary caries, and filling loss. The role of crosslinking agents applied in NaOCl-treated dentin bonding has yet to be experimentally explored clinically.

## 5 Conclusion

Based on the results of this systematic review and meta-analysis, an overall advantage of using collagen crosslinkers in NaOCl treated dentin for enhancing the BS can be observed, which was influenced by the concentration of NaOCl and the duration of exposure. For dentin exposed more than 1 min in NaOCl at concentrations greater than 2.5%, EDTA, PA and SA were observed to significantly improve the bond strength. For dentin exposed less than 1 min or NaOCl at lower concentration, significant positive effect cannot be observed when using collagen crosslinkers. RA proved effective in improving the BS of NaOCl-treated dentin to composite resin at high NaOCl concentrations within a shorter duration. The evidence is insufficient to conclude that IP6 has a positive effect on the bond strength of NaOCl-treated dentin to composite resins.

## Data Availability

The original contributions presented in the study are included in the article/supplementary material, further inquiries can be directed to the corresponding author.
